# The Effect of Different Tightening Torques of Implant Cone Morse Abutment Connection Under Dynamic Fatigue Loading: An In Vitro Study

**DOI:** 10.3390/biomimetics10080511

**Published:** 2025-08-04

**Authors:** Felice Lorusso, Antonio Scarano, Sergio Rexhep Tari, Ishita Singhal, Funda Goker, Maria Costanza Soldini, Gianluca Martino Tartaglia, Massimo Del Fabbro

**Affiliations:** 1 Department of Innovative Technologies in Medicine and Dentistry, University of Chieti-Pescara, 66100 Chieti, Italy; felice.lorusso@unich.it (F.L.); ascarano@unich.it (A.S.); sergiotari@yahoo.it (S.R.T.); 2 Department of Biomedical, Surgical and Dental Sciences, University of Milan, via Della Commenda 10, 20122 Milano, Italy; ishita.singhal@unimi.it (I.S.); gianluca.tartaglia@unimi.it (G.M.T.); massimo.delfabbro@unimi.it (M.D.F.); 3 Unit of Maxillofacial Surgery and Dentistry, Fondazione IRCCS Ca’ Granda Ospedale Maggiore Policlinico, 20122 Milano, Italy; 4 Department of Oral and Maxillofacial Surgery, Faculty of Dentistry, Istanbul Aydın University, 34295 Istanbul, Turkey; 5 Department of Periodontology, Universitat Internacional de Catalunya, 08195 Barcelona, Spain; mariacostanza.soldini@uic.es

**Keywords:** cone morse connection, dental implant, dental implant loading, dental implant–abutment connection, fatigue performance, abutment interface

## Abstract

Background: The implant–abutment joint is important for the long-term marginal tissue integrity in terms of biomimetic design that replicates the natural dentition under mastication forces. This study aimed to evaluate conical implant–abutment joints coupled at different tightening torque values through a mechanical fatigue test. Methods: Eighty conic implants (Ø: 3.8 mm L: 10 mm) with a 6° cone morse joint were embedded in resin blocks with an inclination of 30° ± 2°. The samples were divided into 8 groups (4 Test and 4 Control). The implant–abutment joints were coupled with different tightening torques: 25 Ncm (Group I), 30 Ncm (Group II), 35 Ncm (Group III) and 40 Ncm (Group IV). An in vitro cyclic loading test (1 × 10^4^ loads) was performed for 4 Test groups, while 4 Control groups did not receive any forces. All the samples were assessed with Scanning Electron Microscopy to compare the microfractures and microgaps on flexion and extension points. Results: Microscopy observation results showed significant differences among torque groups. We found that 30 Ncm had the best stability with less microgap. Conclusions: Tightening torque plays an important role in the distortion of the cone morse joint under mechanical forces. However, further studies should be conducted to validate the results using different implant–abutment joints for comparison.

## 1. Introduction

The placement of dental implants is the primary solution for replacing avulsed or missing teeth with high success and survival rates reported in the literature [1,2,3]. Dental implants are important factors in modern restorative dentistry, since they are perfect solutions that can mimic the natural visual appearance and aesthetics, besides the physiological functions as replacements of missing natural teeth. Currently, as a consequence of the increasing number of dental implant insertions, there has been an increase in the incidence of both short- and long-term complications [4,5,6]. These complications can be divided into mechanical or biochemical categories, and all are potential factors for the failure of dental implants and related restorations [7]. Among the long-term complications, dental implant wearing is one of the most frequent ones and can be influenced by several factors including the titanium alloy material, the implant–abutment coupling design, components’ tolerances, microgaps, the preloading forces on screws, oral environment, oral fluids and biomechanical forces. Dental implant wear results in degradation of the surface which can ultimately lead to the failure of the implant or an adverse response to the wear particles. This complex phenomenon can produce alterations in the structural characteristics of the dental implant and can generate strain and fractures which potentially compromise structural integrity. Moreover, the most common implant mechanical failures include fixture body and neck fractures, abutment/joint profile damages and/or deformations, and screw mobility and/or rupture [8]. The main role in such problems is played by the effect of the forces or stress during physiological chewing activities. Parafunctional forces and bruxism can also contribute to a mechanical overload of the implants and generate a higher risk of damage and microfracture [9]. The implant position is another important factor during the different loading patterns of the chewing forces and vectors. In the literature, lateral loading in particular has been considered a determinant role for biomechanical stress and higher levels of marginal bone-loss pattern [9].

The implant–abutment connection is considered as one of the most critical factors for the long-term functional loading and marginal tissue integrity in terms of biomimetic mechanisms and design that replicates the natural dentition under loading mastication forces. The cone morse joint has been described as an implant–abutment connection characterized by a cone-in-cone internal interface [10,11,12]. Biologically, the bacteria colonization can represent a critical factor and the properties of cone morse design seem to reduce the risk of interface penetration when compared to other internal and external connections, as has been mentioned by authors in the scientific literature [10,11]. Furthermore, a cone morse joint with 8° conical angle has been used by the ITI group (International Team for Implantology, Switzerland) to improve the stability of the abutment–implant coupling [13,14]. In the global dental market, different conical implants are available that have different coupling designs, internal conical angles, geometric indexes and interface contact lengths [12]. The main advantage of cone morse joint is determined by the avoidance of marginal microgaps, structural stability and lateral distresses on the fixtures under the loading [15]. In the literature, from the point of periodontal health, cone morse implant was evaluated and as a conclusion it showed a lower level of marginal bone loss at 1 year follow-up compared with internal and external hexagon connections [16]. Moreover, the implant–abutment mechanical coupling is generated through the torquing of an internal screw that contributes to generating the micro-frictional interfaces of the conical profile [8]. However, studies evaluating the outcomes of morse connections torqued with different forces are quite limited. So, it would be important to evaluate the integrity and possible wear of the various implant components in different implant connection configurations, in response to different mechanical stimuli determining the optimal coupling torque of the implant and prosthetic components through an ageing of the components under dynamic fatigue loading. According to a recent systematic review, future simulations in medical investigations using implants are needed in the literature to understand the mechanism regarding abutment–implant connections such as morse tapper or external hexagonal implants because these kinds of publications offer advantages such as lower cost and faster results compared to clinical studies [17]. At this point, this present report is important in that it fills a gap, and it is original because it fixes a lack in information since it concentrates on a specific angle of cone morse connection and also it carries out evaluations with detailed in vitro tests. Therefore, the objective of this study was to examine the mechanical behavior of the 6° cone morse connection at different coupling torque forces of the internal screw through simulation conducted in an in vitro environment. The null hypothesis was that the abutment screw loosening was not influenced by coupling torque implants and fatigue cyclic loading.

## 2. Materials and Methods

For the present in vitro study, a total of 80 implants (10 implants for each group) were evaluated. Four different types of abutment–implant coupling and tightening torques were applied using a calibrated torque meter according to the manufacturer’s protocol. The experiment was conducted in the Department of Innovative Technologies in Medicine and Dentistry of the University “G. d’Annunzio” of Chieti-Pescara.

### 2.1. Dental Implant Characteristics

**Figure 1:**
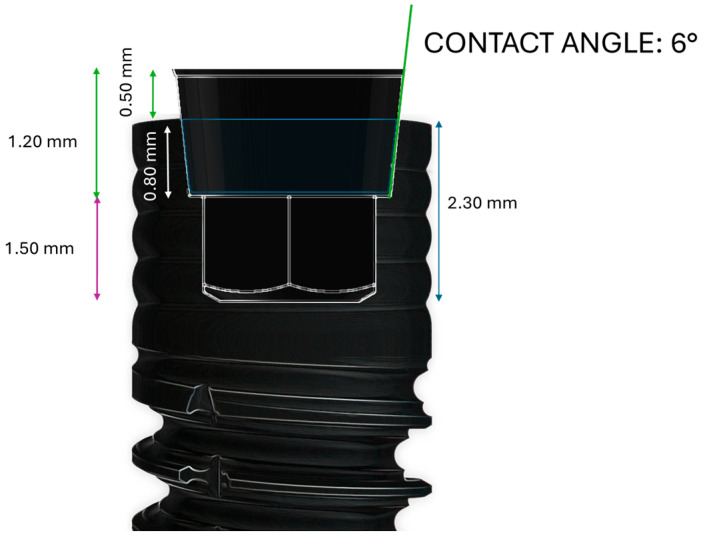
Inner cone morse design of the implants tested in the experiment.

In this experiment, the cone morse dental implant characterized by a tapered shape macro-design was tested. In detail, the most coronal 3/4 part of the implant has a cylinder shape while the apical portion is conical (CLC implant, Vicenza, Italy). The implant–abutment joint is a 6° cone morse with a hexagonal anti-rotational index and switching platform. The 3 mm neck presents a smooth atraumatic multilobed profile and the surface is sandblasted and acid-etched (SLA). This implant joint connection presents double concavity threads and a smooth atraumatic multilobed neck. The implant dimensions used in this study were 3.5 mm diameter and 10 mm length. The implants have sandblasted and acid-etched surface treatment with an average surface roughness of 1.3 μm. The implant microstructure is characterized by a thread pitch of 0.9 mm while the thread extension from the body of the implant is 0.5 mm. The threads present micro-concavities of 0.25 mm that are able to produce a primary distribution on the newly formed bone. The internal chamber conical contact length is 0.80 mm while the total internal chamber length is 2.30 mm. This implant joint connection presents double concavity threads and a smooth atraumatic multilobed neck (Figure 1).

### 2.2. Study Design

**Figure 2:**
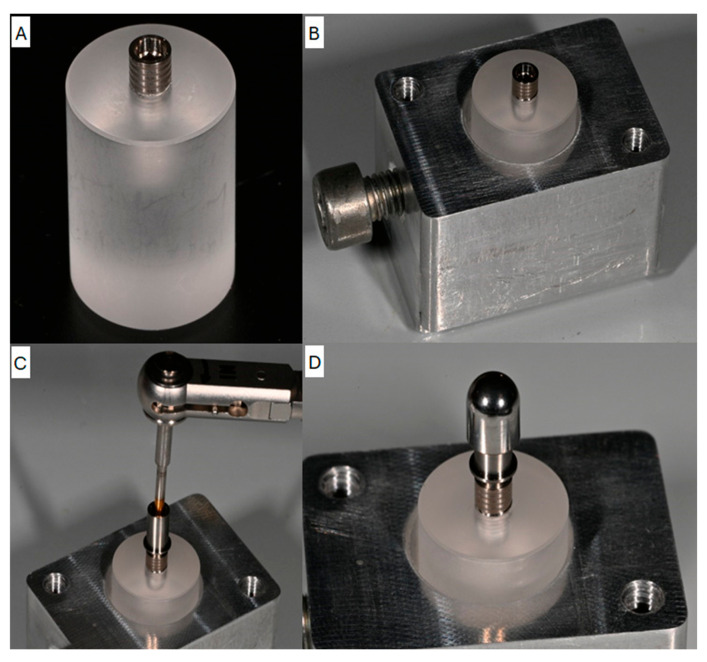
Conic implant that is to be embedded in the resin block. (**A**) The conic implant placed in the resin block. (**B**) The loading cell components. (**C**) Implant–abutment coupling phase. (**D**) Hemisphere cap positioned on the implant–abutment.

The sample size was calculated through the G*Power software package version 3.1.9.7. (University of Stuttgart, Germany) with an effect size of 0.45, α error 0.05 and power 0.80. The estimated sample size was 80 samples (10 implants per group). A total of 80 conic implants (Ø: 3.8 mm L: 10 mm) with a 6° cone morse joint connection were embedded in a resin block with an inclination of 30° ± 2° to reproduce the experimental conditions required with a total of 1 × 10^6^ complete loading cycles (Figure 2A–D).

**Figure 3:**
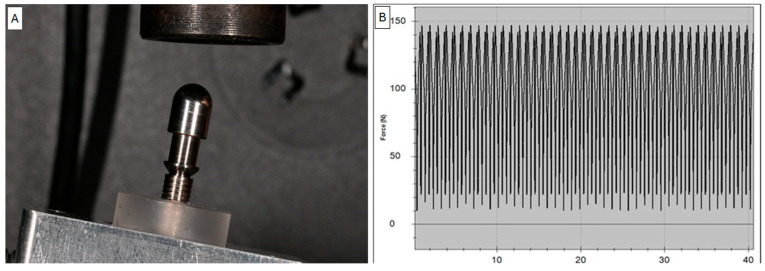
(**A**): A loading angle of 30 degrees was considered for the present test. (**B**) Sample positioned in the loading cell and registration of loading cycles.

The samples were divided into 4 Test and 4 Control groups with 10 implants. For each group, the implant–abutment joints were coupled considering four different tightening torques using a calibrated electronic torque meter (Group I: 25 Ncm; Group II: 30 Ncm; Group III: 35 Ncm; Group IV: 40 Ncm). An in vitro cyclic loading test (1 × 10^4^ loads) was performed for the 4 Test groups while the 4 Control groups did not receive any external forces (Figure 3A,B).

Following is the eight groups described in more detail with respect to the torques applied: Group I-Test and Group I-Control:25 Ncm torque; Group II-Test and Group II-Control:30 Ncm torque; Group III-Test and Group III-Control: 35 Ncm; and Group IV-Test and Group IV-Control: 40 Ncm.

### 2.3. Testing Set-Up

**Figure 4:**
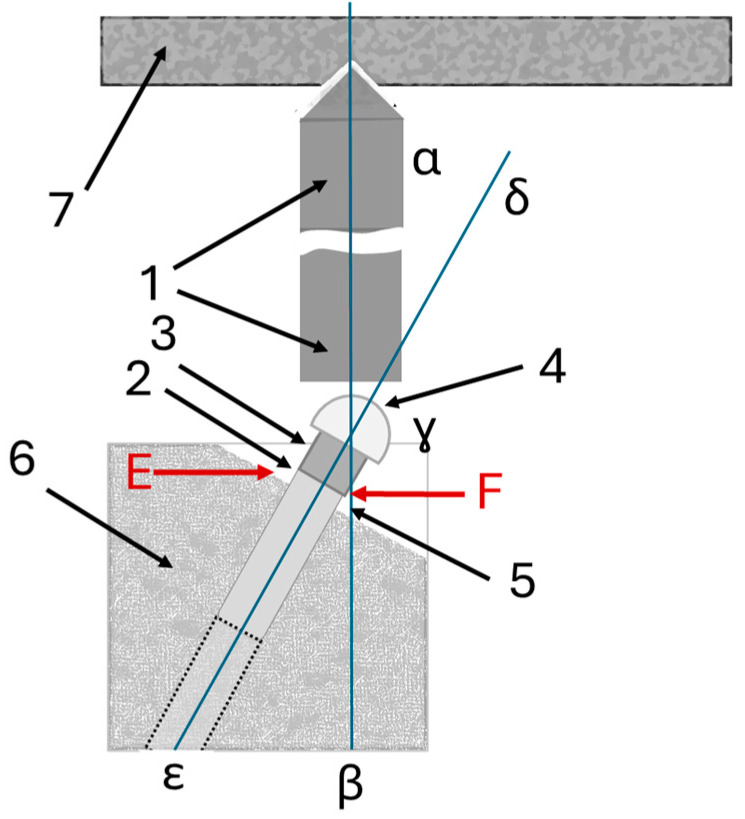
Dental implant embedded in resin cylinder (1 and 7: loading device components; 2, 3, 5: components of the abutment; 2: nominal bone level; 3: supporting part of abutment; 4: resin cylinder; 6: dental implant embedded in resin cylinder; line α-β: direction of the loading force cycles; line ε-γ-δ: inclination of dental implant embedded in resin cylinder with 30° ± 2°; F: flexing; E: extension points).

The experiment was carried out between 1 May 2023 and 1 December 2023. The loading cycles were performed in a controlled environment under constant temperature and humidity conditions (T: 20 ± 5 °C; relative humidity: 65% ± 4%). For the fatigue test, a dedicated single-axis device ZT-HIGH (IMADA, Rome, Italy) for dynamic tests was used with the configuration with a load cell. A dynamic test was considered passed if there were no evident structural defects and/or permanent deformations on the surface of the spherical cap, as well as no signs of failure or wear. The dynamic test was performed under load control with sinusoidal law with an infinite life limit set at 1,000,000 cycles with a maximum stress of F: 88.0 ± 141.0 N (Fmax: 150.0 N and Fmin: 9.0 N). The cycle was considered completed from the pre-load point, maximum peak and the lower loading point. The minimum stress Fmin was 6% of the maximum stress Fmax (Figure 4).

### 2.4. Test Group Loading Test Details

Group I: 25 Ncm. Loading test synthesis—frequency/Hz 3

Group II: 30 Ncm. Loading test synthesis—frequency/Hz 15

Group III: 35 Ncm. Loading test synthesis—frequency/Hz 15

Group IV: 40 Ncm. Loading test synthesis—frequency/Hz 15

### 2.5. Scanning Electron Microscopy (SEM) Analysis

The implants of Test and Control groups were assessed through SEM observations. The samples were embedded in glycol-methacrylate resin (Technovit 7200 VLC, Kulzer, Germany) and upon completion of the polymerization they were sectioned longitudinally from central points with a high-precision diamond disc to obtain thin sections. The microfractures/microgaps between implant and abutment connections on flexing and extension points were assessed and compared with SEM (ZEISS, Oberkochen, Germany). The specimens were also observed using a transmitted light digital microscopy (VHX-7000 4K, Keyence, Osaka, Japan). The morphometric software package for image capturing was used to perform the morphometric assessment using a digital image analysis (VHX-7000, Keyence, Osaka, Japan). The microgaps were calculated at the two peripheral points (right and left points for flexion and extension points at the contact interface) (Figure 4).

### 2.6. Macroscopic Evaluation

The macroscopic evaluation included just the test group which was evaluated with the naked eye for visible damages.

### 2.7. Statistical Analyses

Normality of distributions was evaluated through the d’Agostino and Pearson omnibus test. Variables were not normally distributed, so microgap-measurement-comparison evaluations were carried out as non-parametric tests using paired Mann–Whitney Test versus Control and Wilcoxon matched pairs tests for comparison of flexion (compression) versus extension points. A Kruskal–Wallis test was conducted to compare groups with different torques. *p* = 0.05 was considered significant. The unit of analysis was each implant’s flexing and extension point microgap measurements. Statistical analysis was performed using GraphPad Prism 5.03 (GraphPad Software, Inc., La Jolla, CA, USA).

## 3. Results

### 3.1. Macroscopic Evaluation Results of Test Groups

#### 3.1.1. Group I (25 Ncm)

**Figure 5:**
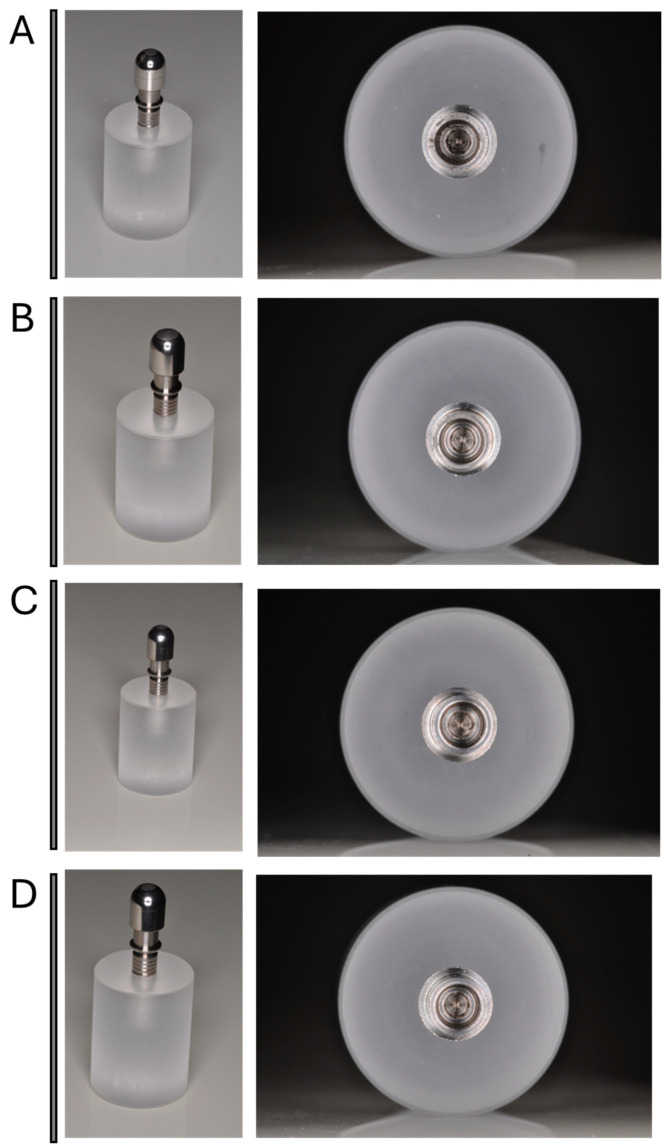
(**A**) Macroscopic details of the implant–abutment joint screw of the Group I post-loading coupling. (**B**) Detail of the implant–abutment joint screw of the Group II post-loading coupling. (**C**) Detail of the implant–abutment joint screw of the Group III post-loading coupling. (**D**) Detail of the implant–abutment joint screw of the Group IV post-loading coupling.

Group I samples were shown to preserve the integrity of the implant–abutment system in response to loading stresses. No failure of the abutment–implant coupling has been reported. There we no signs of alteration of the connection interface. Judging by the observation, there was no evident alteration and/or wear of the implant screw-tightening system (Figure 5A).

#### 3.1.2. Group II (30 Ncm)

Group II samples showed the preserve integrity of the implant–abutment system in response to loading stresses. No abutment–implant coupling failure has been reported for all samples. There were no evident macroscopic signs of deformation of the connection interface. There were no signs of deformation and/or wear of the implant screw-tightening system (Figure 5B).

#### 3.1.3. Group III (35 Ncm)

The implants torqued to the 35 Ncm sample presented the integrity of the implant–abutment system in response to loading stresses. No coupling failure of the implant–abutment joint was reported. There were no macroscopic signs of deformation in the connection interface. On inspection with the naked eye, some tiny marks of damage on the screw head were seen (Figure 5C).

#### 3.1.4. Group IV (40 Ncm)

The Group II samples showed the integrity of the implant–abutment system in response to loading stresses. No abutment–implant system failures were observed. There were no obvious macroscopic signs of deformation in the connection interface. On inspection with the naked eye, some tiny marks of damage on the screw head were seen (Figure 5D).

### 3.2. Microscopic Observation

**Figure 6:**
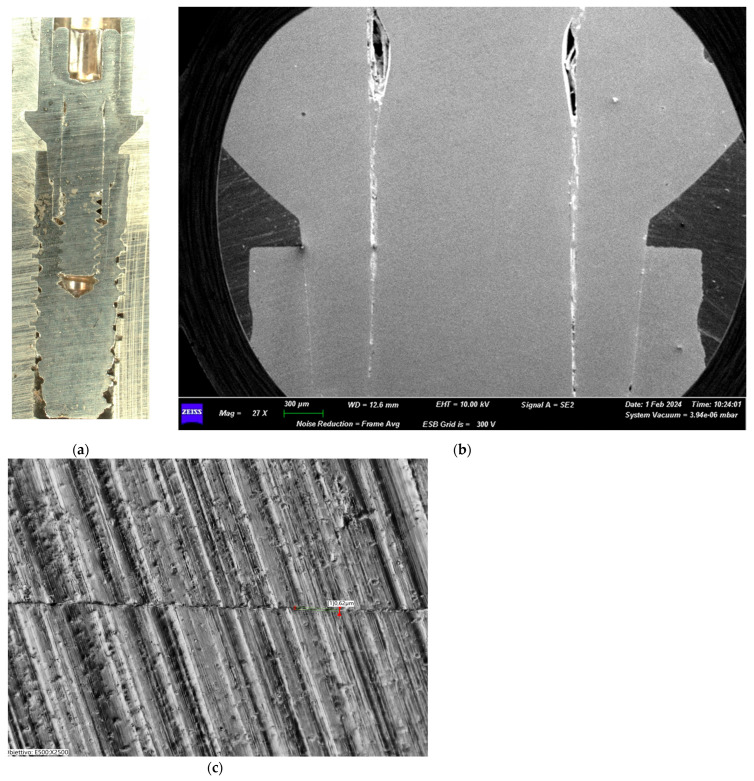
(**a**) Macroscopic section of the implant from Group I. (**b**) SEM image of the implant–abutment joint of Group I (25 Ncm) (magnification: 27×). (**c**) Transmitted light digital microscopy image of the implant–abutment joint microgap of Group I (magnification: 2500×).

The Group I test sample (Figure 6a–c) showed an apparent integrity of the prosthetic connection interface with no signs of deformation lines. No structural damages were evident at the level of the prosthetic chamber interface. Transmitted light microscopy linear morphometry was conducted to measure the implant–prosthetic microgap after the loading.

**Figure 7:**
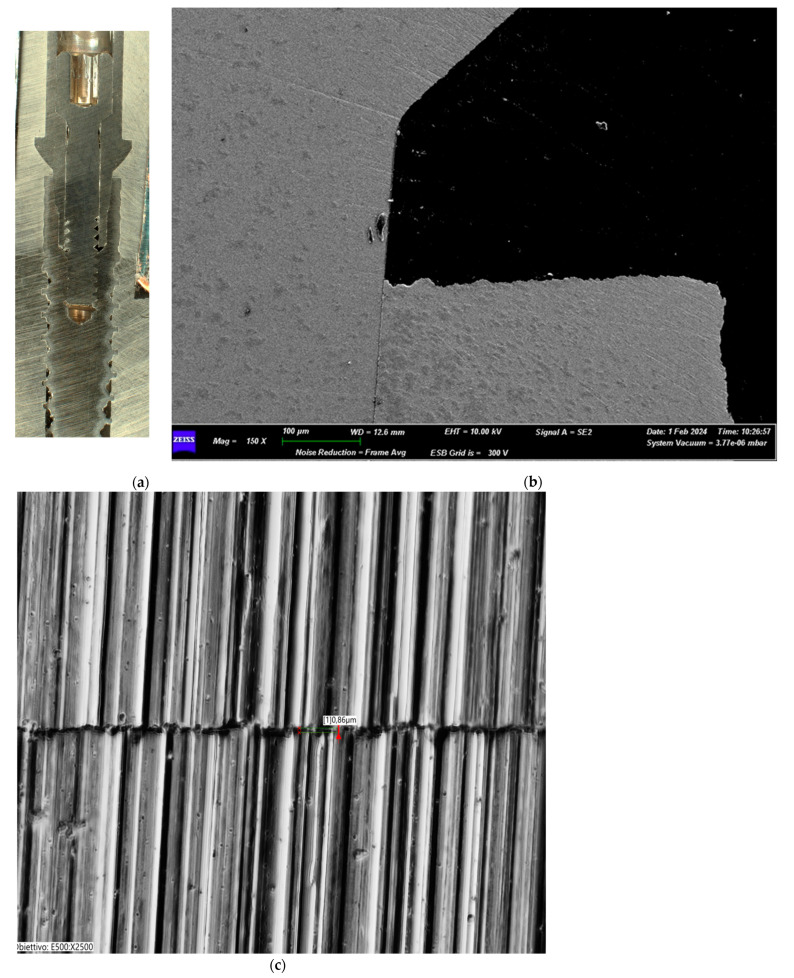
(**a**) Macroscopic section of the implant from Group II. (**b**) Group II (30 Ncm) SEM image of the implant–abutment joint (magnification: 150×). (**c**) Transmitted light digital microscopy image of the implant–abutment joint microgap of Group II (magnification: 2500×).

The Group II test reported no alterations of the prosthetic connection interface with evident signs of optimal coupling of the joint components. No evidence of macroscopic strain lines was present. No structural damages were evident at the level of the prosthetic chamber interface (Figure 7a–c).

**Figure 8:**
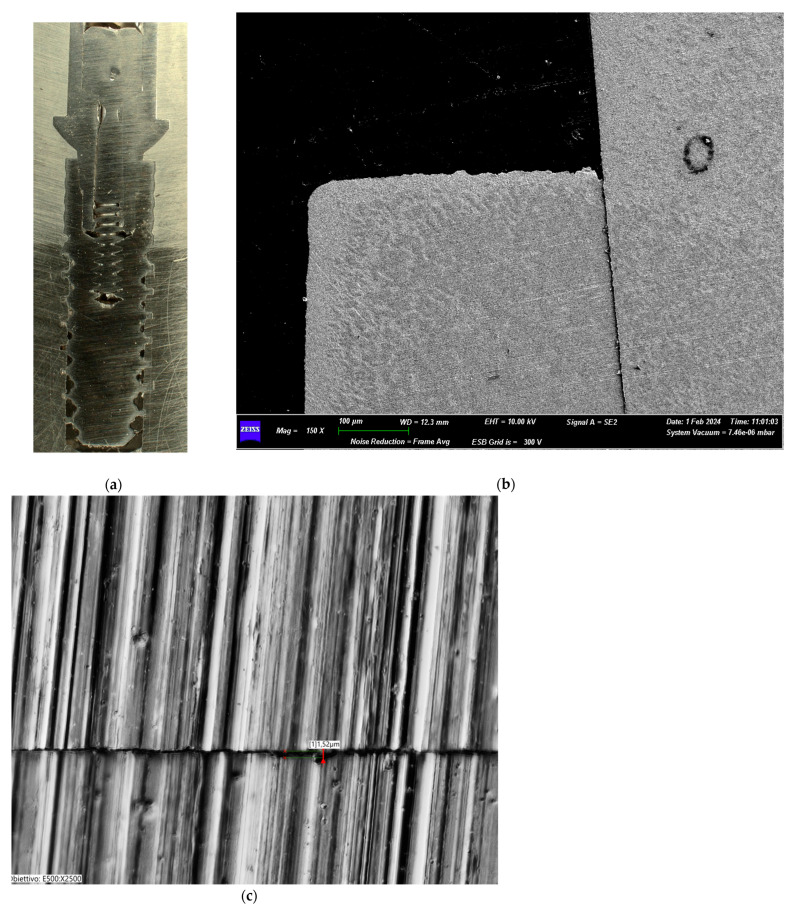
(**a**) Macroscopic section of the implant from Group III. (**b**) Test Group III (35 Ncm) SEM image of the implant–abutment joint (magnification: 150×). (**c**) Transmitted light digital microscopy image of the implant–abutment joint microgap of Group III (magnification: 2500×).

The Group III test sample revealed good integrity of the prosthetic connection interface with no signs of alteration/deformation of the components. At higher magnification, no signs of alteration were present in the most coronal section of the abutment–implant interface. In the deepest section of the connection, the metal microstructure showed some signs of mechanical friction flaking. There were no signs of deformation and/or wear of the implant screw-tightening system (Figure 8a–c).

**Figure 9:**
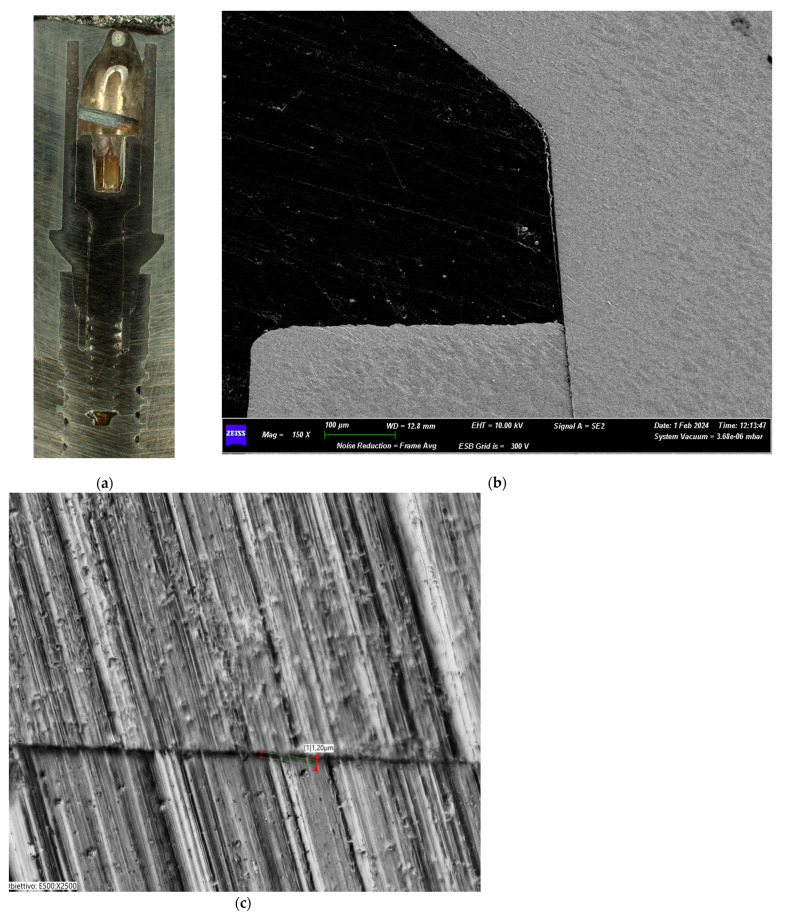
(**a**) Macroscopic section of the implant from Group IV. (**b**) Test Group IV (40 Ncm) SEM image of the implant–abutment joint (magnification: 150×). (**c**) Transmitted light digital microscopy image of the implant–abutment joint microgap of Group IV (magnification: 2500×).

The Group IV test showed an integrity of the joint connection interface with no signs of macroscopic alteration of the components. At higher magnification (250×), there were evident signs of frictional metal flacking of the prosthetic component located in the deep section of the connection, i.e., in the apical region of the angular surface of the abutment. There were no signs of deformation and/or wear of the implant screw-tightening system. No signs of the marginal seal compromissions were evident (Figure 9a–c).

### 3.3. Microgap-Measurement Results

**Figure 10:**
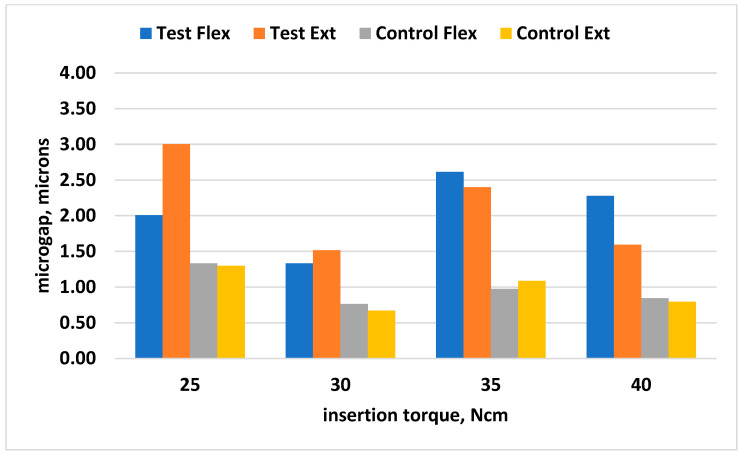
Comparison of torques paired among them for microgaps at extension and flexion points for Test and Control groups.

**Figure 11:**
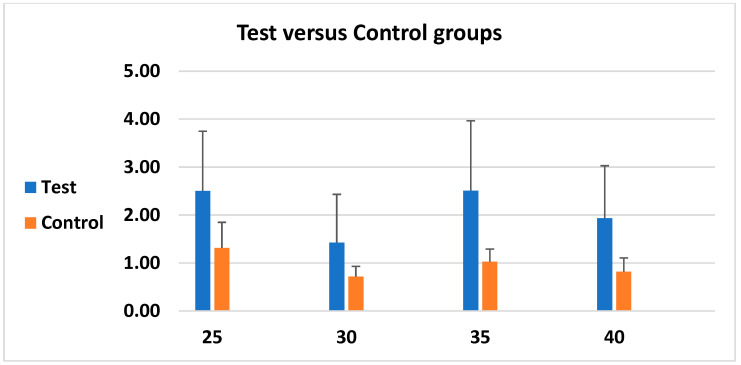
Comparison of Test and Control groups.

In all cases, there was a significant difference among torque groups, which shows the importance of torque. Torque of 30 Ncm had the best stability results with less microgap. When loaded versus non-loaded implants were assessed, the difference was significant as all loaded groups with different torques had less microgap when no load was applied (loading caused more distortion). Further details can be seen in Table 1 and Table 2 and Figure 10 and Figure 11.

## 4. Discussion

The longevity of the implant treatment is one of the critical points in clinical practice. Great efforts have been made over several years of research to formulate clear and conclusive criteria regarding the design, material, surface topology and optimal procedures for dental implant devices. The engineering design and the biomechanics of the implant–abutment connection are some of the priorities when considering the durability of the system [18]. Various authors have studied different types of implant–abutment connections to evaluate and make a comparison among them, as each represents a variety of features with a diversity of advantages and disadvantages (Table 3) [17,19,20,21,22,23,24].

Implant failure may be due to poor oral hygiene, poor bone quality, compromised medical status of the patient and biomechanical factors [25]. Several authors have assessed the importance of biomechanical factors such as type of loading, implant–bone interface, length and diameter of the implants, the shape or characteristics of the implant surface, the type of prosthesis and the quantity or quality of the surrounding bone [25]. Bittencourt et al. investigated microleakage, i.e., the bacterial infiltration between the implant and the abutment. They also evaluated the mechanical behavior concerning the micromovements and the possible loosening or fracture of the screwed abutments, in the conical implant–abutment connections compared to those with internal hexagon, through a fatigue test under cyclic loading [11]. Similarly, this study aimed to evaluate the divergences between the cone morse abutment by applying different coupling torques due to the implications in terms of structural resistance and microfiltration. In more detail, the mechanical behavior of cone morse connection after loading was examined and we tested the application of different coupling torque forces that could potentially produce different effects on interfaces even in the absence of loads. For this purpose, four different controls, one for each coupling force, were adopted to investigate the possible effect.

Several studies indicated that an optimal abutment torque in cone morse implant connection could widely range from 20 to 35 Ncm and it could strictly depend on the coupling design and contact profile [26,27]. Gherke et al. studied through an SEM evaluation an 11° cone morse angle with a contact length of 3.2 mm reporting in vitro the lower microgap value of ~0.3 µm applying 40 Ncm torque [26]. The present study considered a wider torquing range in order to investigate the response of the coupling system to a different set, including potentially non-optimal conditions. Regarding the investigating methods, the purposed technique with SEM represents the best of the knowledge and the state-of-art in this field with respect to measuring the implant–abutment microgap. Although an additional Micro-CT analysis could provide a volumetric evaluation of the inner cone morse chamber, this outcome was out of the scope of the present study. Currently, there is no complete agreement in literature and the alternative evaluation methods would not be alternatives or substitutes but just can be complementary for such implant–abutment interface evaluations.

Analysis of data collected during cyclic loading fatigue testing revealed a significant reduction in the implant–abutment joint microgap. No samples showed interface failure after the loading test. The implant morphometry of the implant torqued at 30 Ncm occasionally produced mechanical friction flaking localized in the internal portion of the joint interfaces with no evidence of sealing defects that appeared to be maintained also at higher tightening coupling. No evidence of coronal marginal involvement was reported in all tested conditions. In the literature, tapered connections demonstrated a greater ability to resist repeated loads over time, maintaining their structural integrity more effectively than internal hexagonal connections [20]. This phenomenon can be attributed to the specific geometry of the conical connection, which favors a more uniform load distribution along the implant–abutment interface. Regarding microleakage, the analysis of leaks around the connections highlighted a lower presence of fluids and bacteria in conical connections compared to the internal hexagonal index [26]. This suggests that tapered connections offer a better seal at the implant–abutment interface, thus reducing the risk of complications such as peri-implant infection and bone loss [27]. Our results are congruent with previous studies that confirmed the advantages of conical connections compared to internal hexagon ones in terms of mechanical stability and resistance to microfiltration. Previous research has also underlined the importance of the seal of the implant–abutment interface in preventing peri-implant complications, confirming the relevance of our results in the context of clinical practice. Implants with a conical connection showed lower levels of microleakage compared to implants with an internal and external connection as well as lower stress at the level of the implants, abutment and crown [28]. Furthermore, it was observed that microleaks decreased with increasing torque [29]. Furthermore, researchers analyzed the degree of adaptation of different implant–abutment connections, both before and after the application of cyclic loads. For this purpose, the samples were sectioned along their longitudinal and transverse axes, and the mismatch of the implant–abutment connection was evaluated using scanning electron microscope analysis. The results indicated that the morse taper connection system showed complete adaptation, characterized by the absence of interstitial spaces after the application of cyclic loads [30]. The findings of our study seem to suggest that microgaps seem to be reduced at 30 Ncm coupling. It is known that the transmission of stresses between the different parts of the implant system is influenced significantly by the connection method adopted. Compared to the convex structure of the external connections, the conical configuration of the internal connections determines a notable increase in the contact areas at the interface between the implant and the abutment. This conical configuration effectively contributes to enhancing the resistance of the abutment screw to lateral forces, while simultaneously improving the flexural resistance capacity of the system. The result of this design is a notable increase in the overall stability and mechanical reliability of the system [31]. Therefore, the use of tapered implants can be promoted as they have better screw stability than other systems. It is critical to consider some limitations of our study, including the simulated nature of the fatigue test and gap analysis under controlled laboratory conditions. Moreover, from a technical point of view the present study investigated coupling interface microgaps, while the loading protocol was not investigated for the dental fracture point and no dental implant failure fractography was performed, since this aspect was not the aim and focus of this experiment. However, further investigations can be carried out to investigate the failure model of cone morse implants under fracture loading tests.

In similar studies, after the application of cyclic loading and intrusion forces, the cone morse joint seems to increase the interlocking interface [19]. Accordingly, a tighter contact between gnd two surfaces seems to occur through juxtaposition until there is no displacement. Therefore, the distribution of chewing forces seems to improve. The greater overlap between the parts seems to cause an increase in implant–abutment interface sealing with more stability [19]. The micromovements between the implant and the prosthetic component can lead to the formation of microgaps at the implant–abutment junction [19]. In this present research, the null hypothesis was that there will be no differences between implant torque coupling, and the study findings seem to support that also the coupling screwing seems to play a significant role for joint stability. The application of 30 Ncm coupling torque seemed to produce the optimal interface with smaller microgaps under fatigue loading.

Implant-based dental rehabilitations represent an increasingly widespread practice for restoring masticatory function following tooth loss. Before being used in a clinical setting, all implant components must pass rigorous laboratory tests to ensure adequate durability. These tests, mainly fatigue tests, are fundamental for evaluating the mechanical performance of systems under simulated loading conditions. The present research aimed to evaluate, through an in vitro simulation, the mechanical behavior of the morse–conical connection coupled at different tightening torque values through a mechanical fatigue test under cyclic loading, as well as to analyze the implant–prosthetic connection interface.

Considering the wide variability of designs present in the global market, the authors chose to test a hexagonal internal indexing and neck profile. This design of a cone morse profile was preferred among alternatives because it is widely used in the world. And the engineering characteristics represented the properties of the implant system tested. Major limitations of this research include small sample size, no comparison group among different inclination angles of loading cycles, no comparison group among different implant–abutment connection types and no comparison among different implant dimensions. Another limitation is the lack of testing using cone morse coupling with different contact angles; however, this was not the aim of this present investigation. As a future perspective, different cone morse designs should be tested.

## 5. Conclusions

According to the results of this in vitro experiment, tightening torque seems to play an important role in the distortion of the cone morse joint that is exposed to mechanical forces. The findings seem to suggest that the microgap seems to be reduced at 30 Ncm torque. However, further research is needed to confirm these results and to evaluate the performance of different implant connections in clinical situations.

## Figures and Tables

**Table 1:** Comparison of results of Kruskal–Wallis test for within Test and Control groups among 25, 30, 35, 40 Ncm torque subgroups.

Kruskal–Wallis Test	Test Flexion	Test Extension	Control Flexion	Control Extension
*p* value	* 0.0121	* 0.0027	** 0.0337	** 0.0021
Significance	*	**	*	**
Do the medians vary significantly (*p* < 0.05)	Yes	Yes	Yes	Yes
Kruskal–Wallis statistic	10.93	14.17	8.693	14.66

* significant; ** highly significant.

**Table 2:** Comparison of torques paired among them for extension and flexion in Test and Control groups.

Dunn’s Multiple Comparison Test	Difference in Rank Sum	Significance	Difference in Rank Sum	Significance	Difference in Rank Sum	Significance	Difference in Rank Sum	Significance
	Test Flexion		Test Extension		Control Flexion		Control Extension	
25 vs. 30 Ncm	9.050	ns	18.1	**	13.95	*	17.65	**
25 vs. 35 Ncm	−7.550	ns	8.85	ns	3.9	ns	2.65	ns
25 vs. 40 Ncm	−3.300	ns	15.25	*	10.15	ns	11.5	ns
30 vs. 35 Ncm	−16.60	**	−9.25	ns	−10.05	ns	−15	*
30 vs. 40 Ncm	−12.35	ns	−2.85	ns	−3.8	ns	−6.15	ns
35 vs. 40 Ncm	4.250	ns	6.4	ns	6.25	ns	8.85	ns

* significant; ** highly significant.

**Table 3:** Comparison of different systems of implant–abutment connections for features, advantages and disadvantages.

Implant–Abutment Connection Types	Properties	Advantages	Disadvantages
Internal connection [23]	Abutments with a connection feature that extends inferior to the coronal portion of the implant	Improved aesthetics Less screw loosening Better microbial seal Better joint strength More platform switching options	The weakest link is the bone rather than the retaining prosthetic screw Less literature on internal connections than external connections
External hexagonal connection [17]	Abutments with an external connection with anti-rotational and indexing features	More scientific literature with long-term follow-up data Compatibility among multiple implant systems Solutions to complications are well investigated due to extensive use	Higher prevalence of screw loosening Higher rates of rotational misfit Fewer aesthetic results Inadequate microbial seal
Platform Switching [21,24]	The diameter of the abutment is narrower than that of the implant. The connection can be both internal and external (more frequently used for internal connection)	Decrease in the stresses around the implant–abutment interface Increase in the forces around the abutment, which results in a decrease of crestal bone loss	Potential lower fracture strength values Increased stress on the abutment and fixation screw The possibility of component maladjustment, screw loosening or fracture Higher costs associated with specialized components to ensure proper component compatibility The learning curve for clinicians is more difficult
Cone screw [20]	Screw with a conical head that allows for a tight fit	Lower marginal bone loss and reduced prosthetic complications	Insufficient tightness under lateral forces (particularly those with shallow taper angles), leading to micromovements and potential bacterial infiltration This can result in higher risk of screw loosening, abutment fracture and marginal bone loss
Morse taper designs [17,19]	Conical connection that involves a trunnion and a bore portion that are both tapered, creating a tight friction fit mechanism. This mechanism occurs when the conical pillar is installed in a conical cavity, generating significant friction due to the parallelism of two structures.	Enhanced stability Reduced micro-movements Therefore, this better fit between the pieces favors joint action, improving the distribution of chewing forces	The potential for abutment fracture and the need for specialized expertise in placement and fitting due to the tight connection
Custom-made abutments [22]	A metal connector (titanium or gold-toned titanium) fabricated by a dental lab to fit an individual’s unique dental implant and occlusion	Custom-made dental implant–abutments offer advantages like superior aesthetics, a more natural feel and improved tissue health	Higher cost and longer processing time compared to stock abutments

## Data Availability

The raw data supporting the conclusions of this article will be made available by the authors on request.
